# Supplementary material for: Perceptual and semantic maps in individual humans share structural features that predict creative abilities
Source: Commun Psychol. 2025 Feb 24;3:30. doi: 10.1038/s44271-025-00214-9 (PMC11850602; doi:10.1038/s44271-025-00214-9)
Supplement: Supplementary file 2 — Additional information [file 44271_2025_214_MOESM2_ESM.pdf]

# Perceptual and semantic maps in individual humans share structural features that predict creative abilities

Johannes P.-H. Seiler[1,*], Jonas Elpelt[2,3,*], Aida Ghobadi[1,*], Matthias Kaschube[2,3,**], Simon Rumpel[1,**]

[1] Institute of Physiology, Focus Program Translational Neurosciences, University Medical Center of the Johannes Gutenberg University Mainz, Duesbergweg 6, 55128 Mainz, Germany

[2] Frankfurt Institute for Advanced Studies, Ruth-Moufang-Straße 1, 60438 Frankfurt am Main, Germany

[3] Institute of Computer Science, Goethe University Frankfurt, Robert-Mayer-Straße 11-15, 60325 Frankfurt am Main, Germany

[*] First authors that contributed equally

[**] Senior authors that contributed equally

Correspondence concerning this article should be addressed to Johannes Seiler or Simon Rumpel, Institute for Physiology, Focus Program Translational Neurosciences, University Medical Center of the Johannes Gutenberg University Mainz, Duesbergweg 6, 55128 Mainz, Germany. E-mail: johseile@uni-mainz.de, sirumpel@uni-mainz.de

- Additional Information -

# Additional information

## Supplementary Figure 1

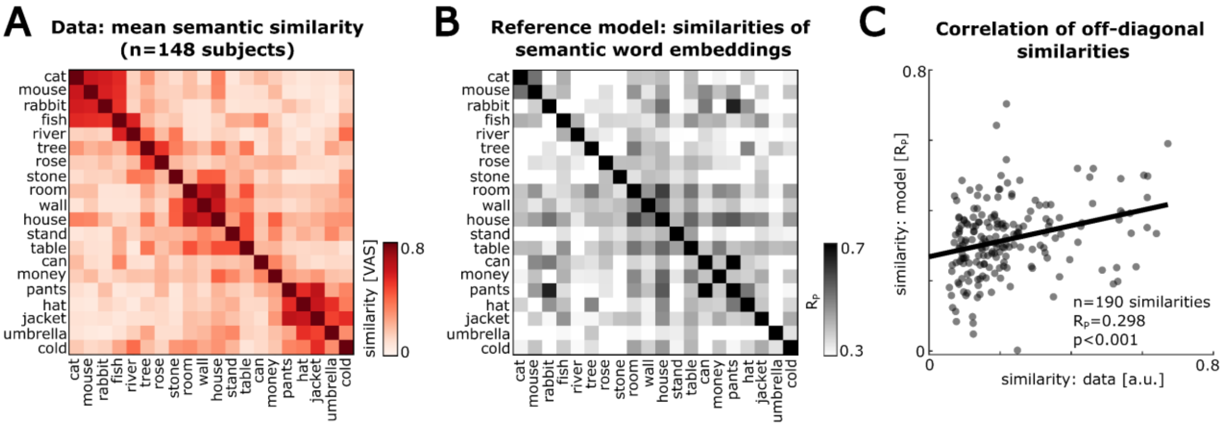


**Supplementary Figure 1 – Comparison of semantic similarity ratings and a reference model of semantic distance**: (**A**) Mean semantic similarity matrix (n=148 participants) as displayed in Figure 1E (VAS: visual analog scale). (**B**) Analogous correlation matrix of semantic word embeddings, using a state-of-the-art open-source multilingual sentence embedding model based on RoBERTa architecture (see Methods). (**C**) Correlation scatter plot of the empirical similarity ratings and the similarities obtained from the word embedding model (n=190 off-diagonal fields from the similarity matrices in A and B). As expected, the empirical similarity ratings are positively associated with the corresponding word embedding similarities.

**Supplementary Figure 2**

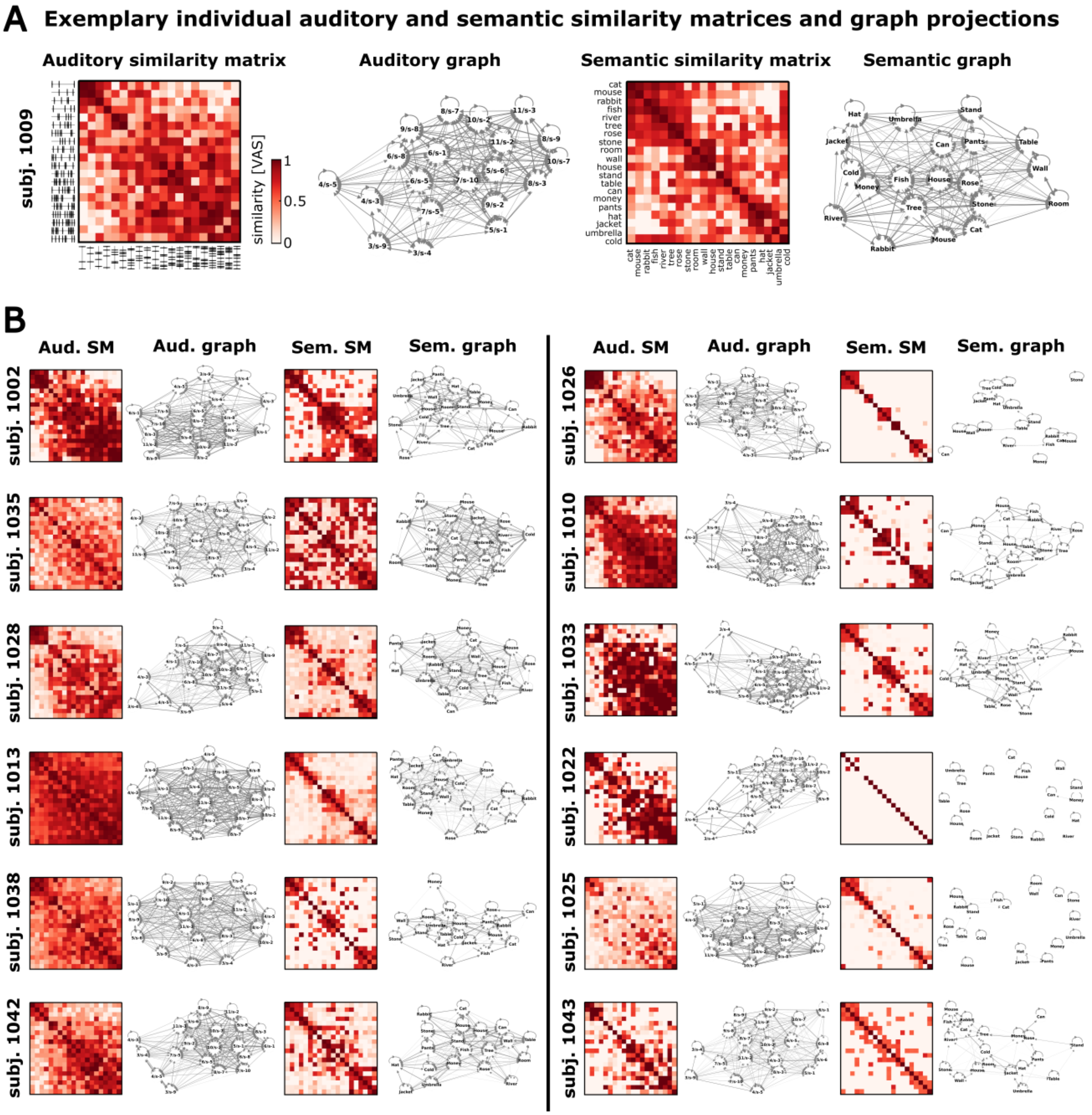


**Supplementary Figure 2 – Exemplary auditory and semantic map estimates from single individuals:** (**A**) Magnified similarity ratings of one exemplary participant from the scaling tasks, displayed as similarity matrix and graph for the auditory and semantic modality. For the graph, only edges with a weight >0 are displayed. (**B**) Additional example participants, showing a variety of individual patterns for the similarity ratings in both modalities.

**Supplementary Figure 3**

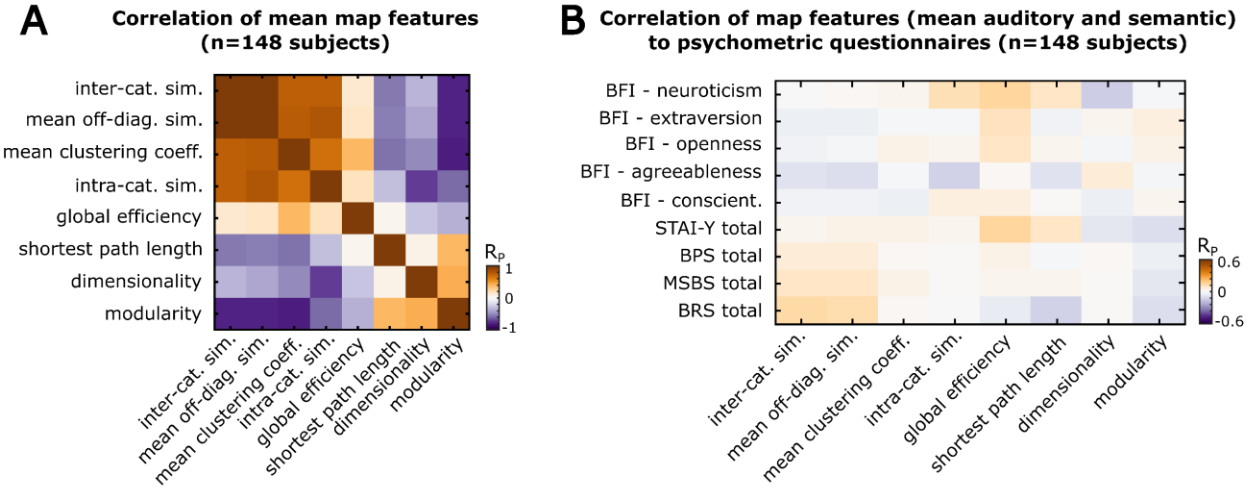


**Supplementary Figure 3 – Correlation patterns of the mean structural map features**: (**A**) Pearson correlation matrix of the mean structural map features, averaged over auditory and semantic modality (n=148 participants). The correlations illustrate the expected relationships between the different map features, covering partially overlapping aspects of representational connectedness, formation of clusters as well as anti-correlated measures of disparity between the mapped stimuli. (**B**) Pearson correlations of the mean map features and the psychometric questionnaire scores (n=148 participants, all correlations: p>0.05). The absence of relevant correlations indicates that the analyzed map features are largely independent from psychometric and psychopathological traits in our sample of healthy students.

**Supplementary Figure 4**

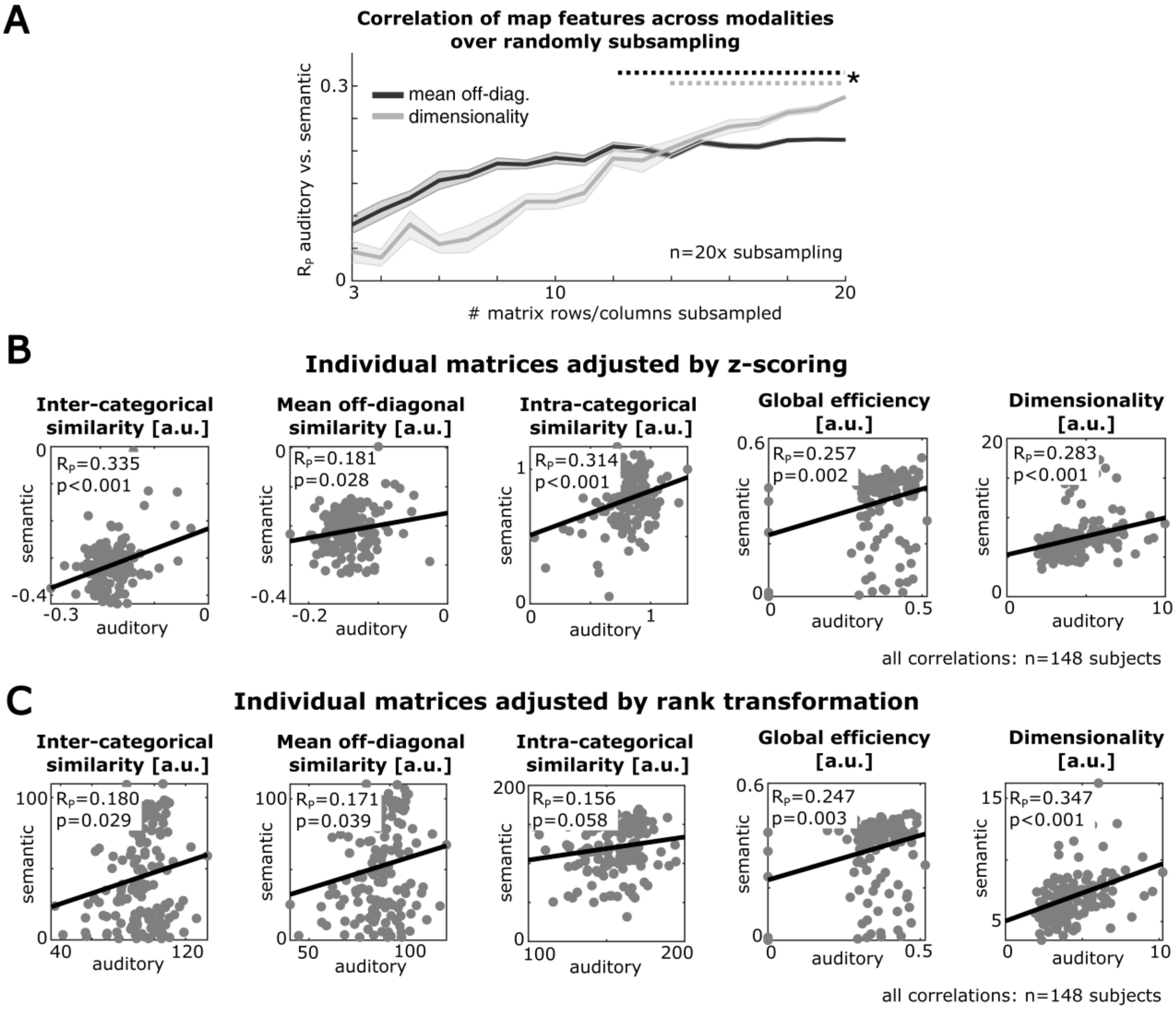


**Supplementary Figure 4 – Controlling the cross-modal correlations of map features for subsampling effects and individual response biases:** (**A**) Cross-modal correlations of two representative map features (mean off-diagonal similarity and dimensionality) over increasing random subsets of the similarity matrices. The positive correlation of auditory and semantic map features develops already for smaller subsamples of the similarity matrices (dashed horizontal bars indicate significant correlations with $p<0.05$ for each map feature). (**B+C**) To control the positive correlation of auditory and semantic map features for individual biases in general response tendencies, we replicated our analyses normalizing each individual's similarity matrix to the subjective dynamic range of responses (z-score, B). as well as by rank-transforming all individual similarity matrices (C) (see Methods for details). The positive correlations of auditory and semantic map features are robust against both corrections for all the different measures.

**Supplementary Figure 5**

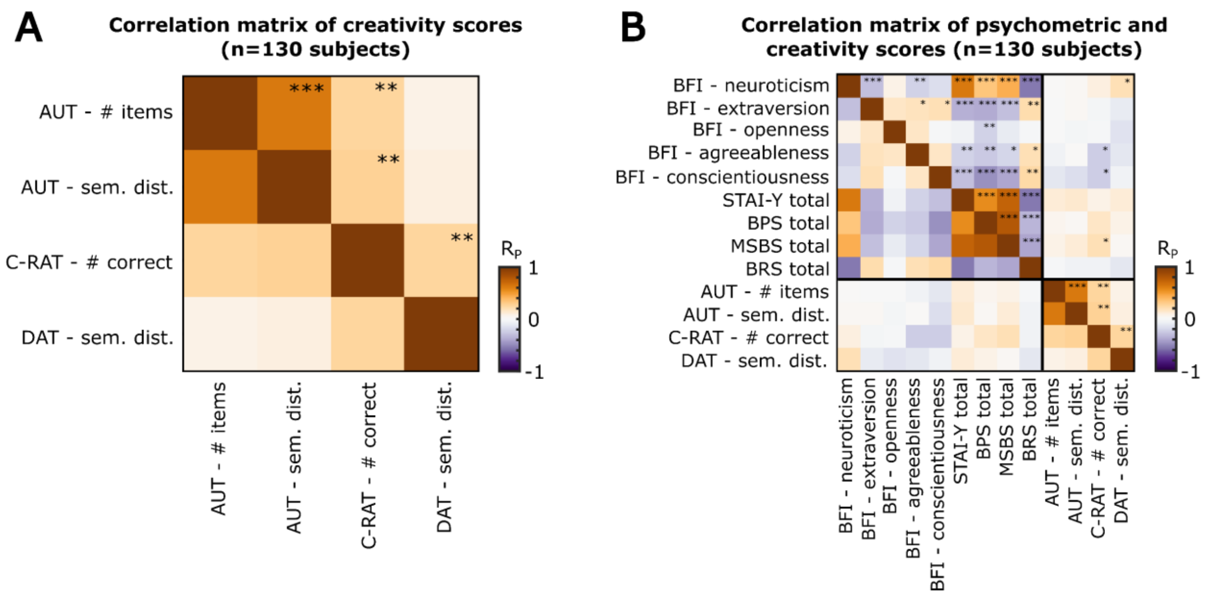


**Supplementary Figure 5 – Correlation patterns of creativity scores and psychometric assessments**: (**A**) Pearson correlation matrix of the different verbal creativity assessments (n=130 participants). As expected, the different measures of divergent thinking, a proxy for creativity, are correlated with each other, where especially the C-RAT shows similarity to the AUT as well as to the DAT (*: p<0.05, **: p<0.01, ***: p<0.001). (**B**) Analogous Pearson correlation matrix including the psychometric questionnaire scores (upper left part of matrix, n=130 participants, *: p<0.05, **: p<0.01, ***: p<0.001. The correlations of A are replicated in the lower right part of the matrix. Creativity assessments and psychometric questionnaires only show weak associations (p-values above significance threshold when correcting for multiple testing). The correlations between the different psychometric scores widely match previous studies, supporting their external validity[57,65].

**Supplementary Figure 6**

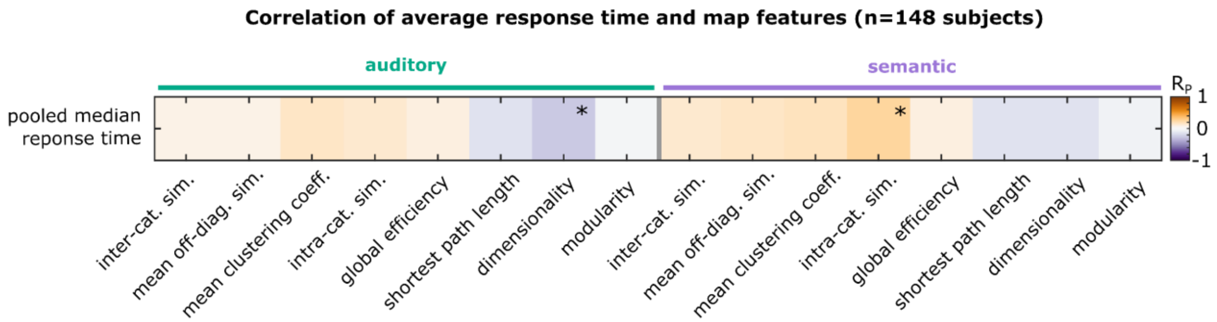


**Supplementary Figure 6 – Average response times only show a minor correlation with representational map features**: (**A**) To estimate the effect of decision-related factors on the representational map estimates in our study, we correlated the median response times of all participants in the scaling task (pooled for both modalities, see Methods) with the extracted map features, we use as a proxy for representational map architectures. Most of the map features did not show a significant correlation with response time. Only auditory *dimensionality* and semantic *intra-categorical similarity* showed a significant, but weak correlation with response times (*: $p<0.003$, indicating statistical significance after Bonferroni correction; all other elements with non-significant correlation). Thus, response times as a proxy for decision-related processes only have a minor effect on representational map estimates in our study, where most map features appear to be largely independent.

**Supplementary Figure 7**

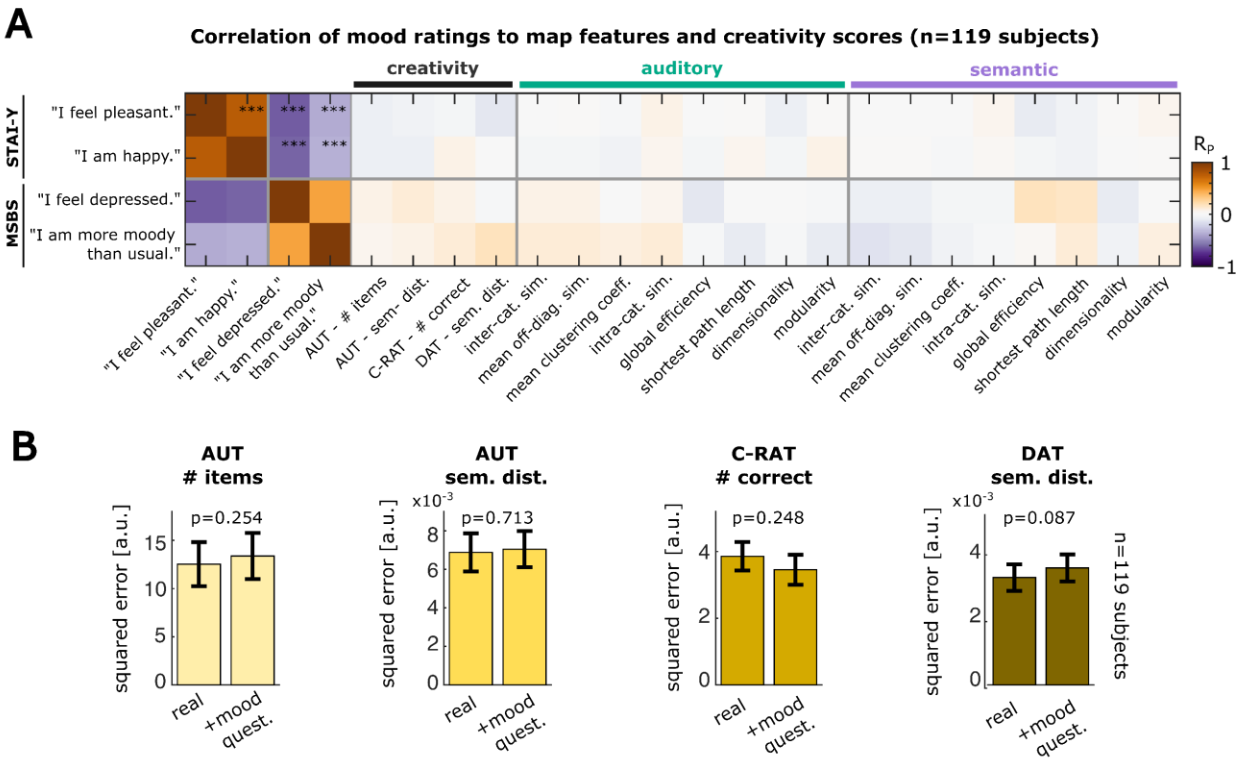


**Supplementary Figure 7 – Self-reported mood does not account for individual representational map features and creativity scores**: (**A**) To test the impact of mood on creativity scores and representational map features in auditory and semantic modality, we evaluated the self-reports on four mood-related items of the STAI-Y and the MSBS (see Methods) and correlated them to the creativity scores and map features of each individual. While the STAI-Y statements, “I feel pleasant” and “I am happy”, assess positive affect directly, the MSBS items, “I feel depressed” and “I am more moody than usual”, assess mood in reverse. All four mood-related ratings were significantly correlated to each other, suggesting consistency and validity in measuring mood. However, we did not observe any significant correlation of the mood assessments and creativity scores or map features (***: $p<0.001$ indicating significance at a Bonferroni-corrected significance threshold of 0.008, all other elements exceed this threshold). (**B**) We also tested if adding the mood-related self-reports to the regression analysis of individual creativity scores (see Figure 3, Methods) improved the predictive power of the regression model. If mood showed a relevant interaction with creativity, we hypothesized that adding the self-reports as regressors would reduce the overall prediction error. Comparing the regression with only the map features (real, equivalent to Figure 3D) against the regression including map features plus mood assessments, we did not find a relevant alteration of the model error for any of the four creativity scores. Together, these analyses demonstrate that the creativity assessments and representational map features in our study are not significantly affected by mood.

**Supplementary Figure 8**

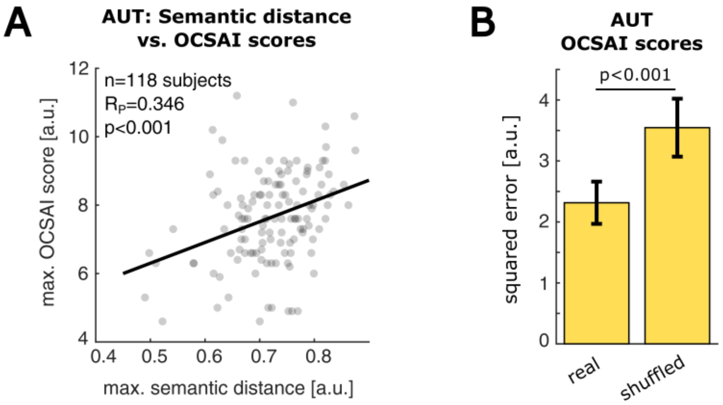


**Supplementary Figure 8 – Qualitative comparability of AUT originality, scored by semantic distance and OCSAI**: (**A**) To evaluate the originality of responses in the Alternative Uses Task (AUT), we relied on an automated scoring method using the maximal semantic distance of responses to the AUT question (see Methods). We compared these semantic distance scores with alternative scores for originality (*OCSAI scores*), based on an evaluation via large language models[40]. Both originality metrics show a significant positive correlation. (**B**) We then replicated our regression analysis of individual creativity scores by each participant's representational map features (see Figure 3D). In line with our results for semantic distance, creativity assessed by OCSAI is significantly predicted by representational map features (all other regression parameters are equivalent to the analyses in Figure 3D). Together, these analyses suggest that semantic distance and OCSAI scores can be used equivalently in our study to assess originality of AUT responses.

**Supplementary Table 1**

| | **Healthy student cohort** (n = 148) |
|---|---|
| **Gender** | |
| Men | 32 (21.6%) |
| Women | 116 (78.4%) |
| **Age** (years) | |
| Mean | 22.7 |
| Standard deviation | 4.9 |
| **BMI** | |
| Mean | 23.8 |
| Standard deviation | 4.4 |
| **Active neuro psychiatric disorder** | |
| No | 148 (100%) |

**Supplementary Table 1 – Demographic characteristics of the study cohort**

**Supplementary Table 2**

| Explained variance ($R^2$) | Refers to: | AUT # items | AUT sem. dist. | C-RAT # corr. | DAT sem. dist. |
|---|---|---|---|---|---|
| **Full map feature data - real** | Fig. 3D, 4B | 0.0255 | 0.0090 | 0.0686 | 0.0108 |
| **Full map feature data - shuffled** | Fig. 3D, 4B | 0.0026 | 0.0026 | 0.0259 | 0.0015 |
| **Modality-general map feature data** | Fig. 4B | 0.0237 | 0.0317 | 0.0185 | 0.0027 |
| **Modality-specific map feature data** | Fig. 4B | 0.0004 | 0.0013 | 0.0146 | <0.0001 |

**Supplementary Table 2 – Explained variance by the different regression models**
