# Supplementary material for: Perceptual and semantic maps in individual humans share structural features that predict creative abilities
Source: Commun Psychol. 2025 Feb 24;3:30. doi: 10.1038/s44271-025-00214-9 (PMC11850602; doi:10.1038/s44271-025-00214-9)
Supplement: Supplementary file 3 — Reporting Summary [file 44271_2025_214_MOESM3_ESM.pdf]

nature portfolio

Corresponding author(s): Johannes P.-H. Seiler

Last updated by author(s): Jan 18, 2025

Reporting Summary

Nature Portfolio wishes to improve the reproducibility of the work that we publish. This form provides structure for consistency and transparency in reporting. For further information on Nature Portfolio policies, see our Editorial Policies and the Editorial Policy Checklist.

Statistics

For all statistical analyses, confirm that the following items are present in the figure legend, table legend, main text, or Methods section.

n/a	Confirmed	
☐	☒	The exact sample size (n) for each experimental group/condition, given as a discrete number and unit of measurement
☐	☒	A statement on whether measurements were taken from distinct samples or whether the same sample was measured repeatedly
☐	☒	The statistical test(s) used AND whether they are one- or two-sided<br>*Only common tests should be described solely by name; describe more complex techniques in the Methods section.*
☐	☒	A description of all covariates tested
☐	☒	A description of any assumptions or corrections, such as tests of normality and adjustment for multiple comparisons
☐	☒	A full description of the statistical parameters including central tendency (e.g. means) or other basic estimates (e.g. regression coefficient) AND variation (e.g. standard deviation) or associated estimates of uncertainty (e.g. confidence intervals)
☐	☒	For null hypothesis testing, the test statistic (e.g. F, t, r) with confidence intervals, effect sizes, degrees of freedom and P value noted<br>*Give P values as exact values whenever suitable.*
☒	☐	For Bayesian analysis, information on the choice of priors and Markov chain Monte Carlo settings
☐	☒	For hierarchical and complex designs, identification of the appropriate level for tests and full reporting of outcomes
☐	☒	Estimates of effect sizes (e.g. Cohen's d, Pearson's r), indicating how they were calculated

Our web collection on statistics for biologists contains articles on many of the points above.

Software and code

Policy information about availability of computer code

Data collection	Custom written MATLAB program (The Mathworks Inc., Natick, Massachusetts, USA, version R2022a).
Data analysis	All analyses were conducted using the MATLAB® statistics and machine learning toolbox (The Mathworks Inc., Natick, Massachusetts, USA, version R2022a) and Python (version 3.8.5; packages: network, scipy, scikit-learn, SentenceTransformers)

For manuscripts utilizing custom algorithms or software that are central to the research but not yet described in published literature, software must be made available to editors and reviewers. We strongly encourage code deposition in a community repository (e.g. GitHub). See the Nature Portfolio guidelines for submitting code & software for further information.

Data

Policy information about availability of data

All manuscripts must include a data availability statement. This statement should provide the following information, where applicable:

- Accession codes, unique identifiers, or web links for publicly available datasets
- A description of any restrictions on data availability
- For clinical datasets or third party data, please ensure that the statement adheres to our policy

The data of this study and the code to analyze it are available via https://doi.org/10.12751/g-node.757evj or from the corresponding authors upon request.

Human research participants

Policy information about studies involving human research participants and Sex and Gender in Research.

Reporting on sex and gender	We used self-report assessments, asking subjects for their gender. We did not specifically assess potential discrepancies between sex and gender. We neither conducted any gender-specific analysis, nor tested for gender differences. Our sample comprised 32 subjects reporting male gender and 116 subjects reporting female gender.
Population characteristics	Apart from the gender distributions, described above, we assessed the age of all participants in our study: mean: 22.7 years, SD: 4.9 years
Recruitment	Healthy students from the University of Mainz were recruited via an online recruiting system to participate in our study (Greiner, 2015, Subject pool recruitment procedures: organizing experiments with ORSEE, Journal of the Economic Science Association). The recruiting system was used to inform subjects about the study and ask them to voluntarily register to participate.
Ethics oversight	The study was registered and approved by the local ethics commitee (Ethikkommission der Landesärztekammer Rheinland-Pfalz, processing number 2024-17477).

Note that full information on the approval of the study protocol must also be provided in the manuscript.

Field-specific reporting

Please select the one below that is the best fit for your research. If you are not sure, read the appropriate sections before making your selection.

☒ Life sciences ☐ Behavioural & social sciences ☐ Ecological, evolutionary & environmental sciences

For a reference copy of the document with all sections, see nature.com/documents/nr-reporting-summary-flat.pdf

Life sciences study design

All studies must disclose on these points even when the disclosure is negative.

Sample size	The sample size for the healthy cohort was estimated based on effect sizes, observed in a pilot experiment, similar to this study.
Data exclusions	A total number of 162 healthy students from the University of Mainz was recruited. From the initial sample, 14 participants encountered technical problems during the experiment, leading to lacking or incomplete data in text ratings and exclusion from our analyses. Thus, a final sample of 148 subjects was used for our analyses. Singtle participants who missed to filled out single items of the psychometric questionnaires were excluded from the analyses of the respective data, leading to deviating numbers of subjects reported for some analyses.
Replication	So far, no replication experiment was conducted.
Randomization	The order of presenting the stimuli in the scaling tasks was randomized. Apart from this, this point is not applicable to our study.
Blinding	Blinding was not relevant in our study. All our analyses were automated to exclude biases due to manual data evaluation.

Reporting for specific materials, systems and methods

We require information from authors about some types of materials, experimental systems and methods used in many studies. Here, indicate whether each material, system or method listed is relevant to your study. If you are not sure if a list item applies to your research, read the appropriate section before selecting a response.

Materials & experimental systems

n/a	Involved in the study	
☒	☐	Antibodies
☒	☐	Eukaryotic cell lines
☒	☐	Palaeontology and archaeology
☒	☐	Animals and other organisms
☒	☐	Clinical data
☒	☐	Dual use research of concern

Methods

n/a	Involved in the study	
☒	☐	ChIP-seq
☒	☐	Flow cytometry
☒	☐	MRI-based neuroimaging
